# Delayed facial nerve decompression for severe refractory cases of Bell’s palsy: a 25-year experience

**DOI:** 10.1186/s40463-017-0250-y

**Published:** 2018-01-04

**Authors:** Ilyes Berania, Mohamed Awad, Issam Saliba, Jean-Jacques Dufour, Marc-Elie Nader

**Affiliations:** 1 Division of Otolaryngology - Head and Neck Surgery, Université de Montréal, Centre Hospitalier de l’Université de Montréal (CHUM) – Hôpital Notre-Dame, 1560 Sherbrooke Street, Montreal, QC H2L 4M1 Canada; 2 Department of Head and Neck Surgery, Unit 1445, The University of Texas MD Anderson Cancer Center, 1515 Holcombe Blvd, Houston, TX 77030 USA

**Keywords:** Bell’s palsy, Surgical decompression, Facial nerve, Functional outcomes

## Abstract

**Background:**

This study aims to assess the effectiveness of delayed facial nerve decompression for Bell’s palsy (BP).

**Methods:**

We performed a retrospective case review of all patients having undergone facial nerve decompression for severe refractory BP between 1984 and 2009 at our tertiary referral center. Demographics, timing between onset of symptoms and surgical decompression, degree of facial nerve dysfunction pre- and post-operatively, follow-up length after surgery and postoperative complications were recorded. Facial nerve dysfunction was assessed using the House-Brackmann (HB) scale. Electroneuronography, electromyography and imaging results were assessed when available.

**Results:**

Eighteen patients had surgery between 21 and 60 days after onset of BP (group I), and 18 patients had surgery more than 60 days after onset of symptoms (group II). In group II, 11 patients had surgery between 61 and 89 days and 7 patients after 90 days. Groups I and II showed similar functional gain and rates of improvement to HB 3 or better (11/18 vs. 11/18, *p* > 0.05). In group II, patients operated 60 to 89 days after onset of BP showed a significantly higher rate of improvement to HB 3 or better (9/11 vs. 2/6, *p* = 0.049) with higher functional gain compared to those operated after 90 days (*p* = 0.0293).

**Conclusions:**

When indicated, facial nerve decompression for BP is usually recommended within the first 2 weeks of onset of facial paralysis. Nonetheless, our results suggest that patients with severe BP could benefit from decompression surgery within 90 days after onset of symptoms in the absence of an opportunity to proceed earlier to surgery. Further investigation is still required to confirm our findings.

**Trial registration:**

Retrospective registered. IRB# 2016–6154, CE 15.154 – CA

## Background

Idiopathic facial paralysis, also defined as Bell’s palsy (BP), is the most common peripheral mono-neuropathy affecting approximately 20–30 per 100,000 individuals annually [1]. This condition causes an acute dysfunction of the facial nerve, which may be partial or complete. The disorder is typically unilateral and affects mainly voluntary facial muscle contraction [2]. Motor dysfunction may lead to incomplete eyelid closure, predisposing to corneal abrasion, exposure keratitis or corneal ulcerations [3]. Patients with Bell’s palsy may also complain of xerostomia, dysgeusia and aural pain. These symptoms seem associated with a poorer nerve recovery prognosis [4]. Although most cases are self-limited, about 4% of patients remain with severe and persistent facial nerve dysfunction [5].

While there is strong evidence for initial conservative medical treatment in patients with BP [6–8], surgical decompression of the facial nerve remains a more controversial subject. Varying opinions exist in regards to the optimal surgical approach, extent of nerve decompression and timing of surgery. Both, the transmastoid and middle fossa approaches have their proponents. Some authors have argued that the transmastoid approach is an effective treatment option that allows sufficient access to the facial nerve with low complication rates [9]. They have described techniques to decompress the geniculate ganglion and the distal labyrinthine segment while avoiding temporal lobe compression. Others have abandoned that procedure in favor of the middle fossa approach. They believe it offers better access to the facial nerve medial to the geniculate ganglion while having an acceptable complication risk profile [10]. The controversy around the choice of the surgical approach is closely related to the debate regarding the extent of nerve decompression. Some authors have recommended to decompress specifically the labyrinthine and meatal segments of the facial nerve [10]. These recommendations are based on studies showing that these two segments are the narrowest portions of the temporal bone at which nerve conduction blockage commonly occurs [11, 12]. Others have recommended instead subtotal decompression from the labyrinthine segment to the stylomastoid foramen based on their results showing reduced recurrences of BP and improved recovery [13]. Regarding timing of surgery, it is generally accepted that most favorable outcomes are obtained when decompression is performed within the first 14 days after onset of symptoms [14]. However, some studies have suggested potential benefits from delayed decompression anywhere from 1 month up to 4 months following the onset of BP [9, 15, 16]. Determining the role of delayed decompression is clinically relevant, especially considering the reality of the Canadian Healthcare system where patients may experience significant delays before being assessed by a neurotologist.

The benefits of surgical decompression in BP still need to be further clarified because of the lack of randomized reports addressing the subject and the potential significant surgical risks. More precisely, the role of delayed surgical decompression remains unclear. The present study reports clinical outcomes of patients with persistent and severe Bell’s palsy who underwent delayed facial nerve decompression surgery at our institution.

## Methods

After obtaining institutional review board approval (University of Montreal Hospital Center, IRB# 2016–6154, CE 15.154 – CA), we conducted a retrospective case review that included all patients having undergone facial nerve decompression between 1984 and 2009 for severe refractory BP at our tertiary care center. A total of 36 patients were selected based on the following inclusion criteria: 1) Early onset facial paralysis concordant with a diagnosis of Bell’s palsy, 2) age ≥ 18 years, 3) patients with a persistent House-Brackmann (HB) grade V or VI in early assessments prior to surgical intervention, 4) initial management with high-dose steroids with or without antiviral agents or clinical surveillance only (i.e. no oral steroids).

Patients presenting early, within 2 weeks of onset of BP, were treated medically with oral corticosteroids. Those diagnosed within 3 days of onset also received antiviral therapy. Surgical decompression was systematically offered to patients with persistent facial paralysis HB grade 5 or 6 who showed more than 90% denervation on electroneuronography (ENoG). A certain proportion of our patients could not undergo ENoG testing, either because they presented more than 21 days after onset of symptoms or testing was unavailable. It was the practice of the senior neurotologist (JJD) to offer surgery to those patients after an in-depth discussion about the controversies and uncertainty of this treatment option in the absence of electrophysiological testing.

All 36 patients underwent transmastoid decompression surgery. A surgical technique similar to the one described by Yanagihara et al. was followed [17]. In summary, after completing a mastoidectomy, the vertical segment of the facial nerve was identified, and the facial recess was drilled out. The incudostapedial joint was carefully disarticulated, and the incus was temporarily removed. Air cells anterior to the superior bony semicircular canal were widely dissected, allowing access to the geniculate ganglion and distal labyrinthine segment. Through this transmastoid approach, the facial nerve was decompressed 180 degrees from the distal labyrinthine segment to the stylomastoid foramen, including the geniculate ganglion. The epineurium was carefully incised using a fine Beaver blade. At the end of the procedure, a tympanomeatal flap was elevated, and the incus was interposed. Two patients also underwent a middle fossa exposure in addition to a transmastoid approach. This change in surgical technique reflected the individual preference of one of the neurotologists (IS) who joined our institution in 2003.

Patients who underwent surgical decompression were classified according to the time between the onset of facial paralysis, determined during the first visit with a neurotologist, and day of the surgery; group I included patients who had surgery 21 to 60 days after onset of symptoms while group II comprised those with surgery performed >60 days. The second group was further divided as group IIa for patients having undergone surgery between 61 and 89 days and group IIb for those operated 90 days or more after onset of symptoms. Our study did not identify any patients who had early surgery within 21 days of initial diagnosis.

For each patient, the following data was obtained: demographic parameters, initial use of conservative therapy, timing between onset of symptoms and surgical decompression, degree of facial nerve dysfunction pre- and post-operatively, follow-up visits after surgery, and postoperative complications. Facial nerve dysfunction was assessed using the House-Brackmann (HB) grading scale. Electroneuronography, EMG and imaging results were assessed when available.

Functional improvement of facial nerve was reported using three methods: 1) Proportion of patients with a final HB grade of 3 or better; 2) Simple functional gain was defined as the algebraic difference between pre and postoperative HB grades; 3) Weighted functional gain relied on a point-based scale more reflective of the clinical function of the facial nerve. It was based on postoperative HB grades (Table 1), and it allowed us to give more importance to HB 1 and 2 results compared to the simple functional gain method. Also, as opposed to the simple function gain calculation, no points were given if HB scores went from 6 to 5, as this degree of change did not represent a clinically significant improvement.

**Table 1:** Weighted functional gain scale

Pre and post-operative HB score	Improvement points attributed
6 to 6	0
5 to 5	0
6 to 5	0
5 to 4	1
6 to 4	1
6 to 3	2
5 to 3	2
6 to 2	3
5 to 2	3
6 to 1	4
5 to 1	4

### Statistical analysis

All statistical analyses were performed using SPSS version 19.0 (SPSS, Chicago, IL, USA). Data presented as ratios was analyzed using the Pearson χ^2^ test or Fisher exact 2-tailed test if there were fewer than 10 patients in any cell of a 2 × 2 grid. Parametric demographic and clinical data were analyzed using the ANOVA and t-test. Simple and weighted functional gains were evaluated using the Mann-Whitney test as these represented non-parametric data. Correlation between the length of follow-up and facial nerve improvement was evaluated using linear regression analysis. A *p* value <0.05 was considered statistically significant.

## Results

Our studied population included 19 males (52.7%) and 17 (47.2%) female patients. The mean age at diagnosis was 47.0 (± 14.4) years. Three (8.6%) patients had a recurrent condition at the time of assessment. Among our group of patients, 26 (72.2%) patients presented total facial paralysis (HB VI). ENoG studies were available for 13 (36.1%) patients, all of whom had 0% residual nerve function on testing. Follow-up visits of patients were between 30 days and 8 years post-operatively. A mild correlation was noted between length of follow-up and improvement in facial nerve function when using final HB grade (R squared = 0.31, *p* < 0.01) and weighted functional gain (R squared = 0.30, p < 0.01) as our measures of facial nerve improvement. No correlation was noted between length of follow-up and simple functional gain. (R squared = 0.06, *p* = 0.1454). Additional parameters are shown in Table 2.

**Table 2:** Demographic data and clinical characteristics of patients

Characteristic	Total (*n* = 36)	Decompression, 21–60 days (*n* = 18)	Decompression, 60–89 days (*n* = 11)	Decompression, ≥ 90 days (*n* = 7)	*P* value
Age (mean ± SD)	47.0 ± 14.4	46.0 ± 12.9	47.7 ± 17.1	50.6 ± 16.3	*P* = 0.767
Male/female ratio	19/17	9/9	7/4	3/4	*P* = 0.6529
Median months of follow-up (range)	12.2 (1.0–97.3)	7.7 (1.0–24.3)	17.6 (1.0–97.3)	15.0 (2.0–84.0)	*P* = 0.164
Affected side (Right/Left)	17/19	9/9	4/7	4/3	*P* = 0.6529

*SD* standard deviation

Our study population included 18 (50.0%) patients who underwent surgery 21 to 60 days (group I) and 18 (50.0%) patients who had surgery >60 days (group II) after onset of symptoms. Within group II, 11 (61.1%) cases underwent surgical decompression between 60 and 89 days (group IIa) and 7 (38.9%) patients after 89 days (group IIb). Post-operative clinical improvement to HB 3 or better at the last follow-up visit was observed for 11 (61.1%) patients in group I and 11 (61.1%) patients in group II. In the second group, 9 (81.8%) patients in group IIa and 2 (28.6%) patients in group IIb, showed favorable recovery (*p* = 0.049). Additional parameters including final HB grading and preoperative studies are available in Table 3.

**Table 3:** Pre- and postoperative clinical parameters

Parameters	Total (*n* = 36)	Decompression, 21–60 days (*n* = 18) (Group I)	Decompression, 60–89 days (*n* = 11) (Group IIa)	Decompression, ≥ 90 days (*n* = 7) (Group IIb)	*P* value
Patients treated with preoperative steroids (no, %)	19 (52.8)	11 (61.1)	6 (54.5)	2 (28.6)	*P* = 0.4130
Preoperative EMG (no, %)	11 (30.6)	5 (27.8)	4 (36.4)	2 (28.6)	*P* = 0.8810
Preoperative ENoG (no, %)	13 (36.1)	7 (38.9)	4 (36.3)	2 (28.6)	*P* = 0.8900
Delay before surgery (Mean days, range)	63 (21–205)	38 (21–57)	68 (62–79)	127 (90–205)	
Initial Facial Function (HB mean, ± SD)	5.7 ± 0.6	5.7 ± 0.6	5.6 ± 0.7	5.7 ± 0.5	*P* = 0.5200
Final Facial Function (HB mean, ± SD)	3.3 ± 0.9	3.2 ± 0.7	2.9 ± 1.0	4.0 ± 0.8	*P* = 0.0772
Simple Functional Gain (mean gain, ± SD)	2.4 ± 1.0	2.4 ± 1.0	2.8 ± 0.8	1.7 ± 1.1	#*P* = 0.0293
Weighted Functional Gain (mean gain, ± SD)	1.7 ± 0.9	1.7 ± 0.8	2.1 ± 1.0	1.0 ± 0.8	*P = 0.0314
Final HB score 3 or better (no, %)	22 (61.1)	11 (61.1)	9 (81.8)	2 (28.6)	**P = 0.049
Final HB score 2 or better (no, %)	6 (16.7)	3 (16.7)	3 (27.3)	0 (0)	*P* = 0.3531

*No* number

*%* percentage

*SD* standard deviation

*HB* House-Brackmann score

#Statistical significance, simple functional gain between Group IIa (decompression 60–89 days) and Group IIb (≥ 90 days) in Mann-Whitney test (*p* = 0.0293)

*Statistical significance, weighted functional gain between Group IIa (decompression 60–89 days) and Group IIb (≥ 90 days) in Mann-Whitney test (*p* = 0.0314)

** Statistical significance, final HB score 3 or better between Group IIa (decompression 60–89 days) and Group IIb (≥ 90 days) in Fisher exact test (*p* = 0.049)

Comparison of clinical improvement scores revealed no significant difference between surgical decompression in patients who underwent surgery 21 to 60 days (group I) compared to patients operated after 60 days (group II, *p* = 0.9862). However, we noted that patients who underwent surgical decompression between 60 and 89 days (group IIa) showed a statistically significant higher clinical improvement score in comparison to those operated past 90 days (group IIb) for simple functional gain and weighted functional gain (*p* = 0.0293 and *p* = 0.0314, respectively) (Table 3).

We performed an additional subgroup analysis of only those subjects who had been initially managed with oral steroids. This analysis also excluded the two patients who underwent middle fossa decompression as to have a more homogenous population. No significant difference in clinical improvement scores was noted between group I (11 patients) and group II (eight patients) (Table 4).

**Table 4:** Pre- and postoperative clinical parameters of patients initially treated with oral steroids

Parameters	Total (*n* = 19)	Decompression, 21–60 days (*n* = 11) (Group I)	Decompression, >60 days (*n* = 8) (Group II)	*P* value
Preoperative EMG (no, %)	5 (26.3)	3 (27.3)	2 (25.0)	*P* = 0.9116
Preoperative ENoG (no, %)	6 (31.6)	4 (36.4)	2 (25.0)	*P* = 0.5988
Delay before surgery (mean days, range)	55 (24–113)	40 (24–57)	76 (62–113)	
Initial Facial Function (HB mean, ± SD)	5.9 ± 0.3	6.0 ± 0.0	5.7 ± 0.5	*P* = 0.3681
Final Facial Function (HB mean, ± SD)	3.2 ± 0.7	3.2 ± 0.8	3.3 ± 0.7	*P* = 0.9045
Simple Functional Gain (mean gain, ± SD)	2.5 ± 1.0	2.5 ± 1.1	2.4 ± 0.8	*P* = 0.8103
Weighted Functional Gain (mean gain, ± SD)	1.7 ± 0.8	1.7 ± 0.9	1.8 ± 0.7	*P* = 1.0
Final HB score 3 or better (no, %)	12 (63.2)	7 (63.6)	5 (62.5)	*P* = 0.9596
Final HB score 2 or better (no, %)	3 (15.8)	2 (18.2)	1 (12.5)	*P* = 0.7374

*No* number

*%* percentage

*SD* standard deviation

*HB* House-Brackmann score

The most commonly encountered surgical complication was tympanic membrane perforation following tympanomeatal flap elevation in 4/36 patients (11.1%). The other reported complications were post-operative hematoma (2/36, 5.6%) and surgical site infection (1/36, 2.8%). Audiogram results were not systematically available, and no conclusions could be drawn regarding the rate of hearing loss. Review of the operative reports did not reveal any instances of damage to the bony labyrinth or increased difficulty decompressing the distal labyrinthine segment of the facial nerve.

## Discussion

To our knowledge, the present study represents one of the largest assessments of patients who underwent delayed surgical decompression for severe BP refractory to medical treatment. While no significant differences were noted between the two main comparison groups, the subgroup analysis suggests that patients with severe BP may benefit from decompression surgery up to 90 days after the onset of symptoms in the absence of an opportunity to proceed earlier to surgery.

Previous studies have reported variable results following delayed facial nerve decompression in patients with refractory BP. Similar to our findings, Bodenez et al. have reported favorable outcome with delayed facial nerve decompression in 13 patients with advanced facial paralysis who were operated between 1 to 4 months from the onset of BP [15]. At a one-year follow-up assessment, all their patients had showed clinical recovery to HB grade III or better. Yanagihara et al. reached similar conclusions [9]. They reported outcomes following transmastoid decompression done between 15 and 120 days after the onset of BP. Although they noted optimal results when surgery was performed early, their data suggested that delayed decompression up to 3 months may still be beneficial. They noted that 38.1% of patients operated between 31 to 60 days after onset achieved HB grade 1 compared to only 23.2% in the control group. Also, all patients treated after 60 days achieved a score of HB 3 or better compared to 86% in the nonsurgical group. On the other hand, other studies have not found delayed decompression to be as effective. Li et al. examined the outcome of transmastoid decompression 2 months after the onset of symptoms in refractory BP patients who were initially treated with corticosteroids [18]. Patients who underwent surgery between 2 and 3 months after onset of symptoms had an initial higher rate of facial function improvement compared to the control group at the three-month follow-up. However, that difference was not present at the 12-month follow-up visit. Similarly, Kim et al. evaluated the effectiveness of delayed transmastoid facial nerve decompression between 3 weeks and 2 months in 12 patients with BP [19]. Their study did not demonstrate any significant difference in facial nerve function between the surgical and control groups. Another study has recently reported recovery outcomes following delayed surgery done 2 weeks after diagnosis for patients with facial paralysis not responding to conservative management. It showed better functional gain when nerve decompression was performed within 2 weeks of onset of BP compared to delayed surgery beyond 26 days or medical treatment alone. Some beneficial effects were observed when surgery was done between 15 and 25 days [16]. Finally, Gantz et al. found greater recovery rates to HB grade I-II from facial paralysis if surgery was performed within 14 days of onset of BP [14]. Except for the study by Li et al., the other papers did not include a subgroup analysis comparing patients who underwent surgery beyond 90 days to those operated 14 to 90 days after onset of symptoms. Therefore, findings similar to ours may not have been identified in these studies because of this absence of subgroup analysis.

The current clinical practice guidelines strongly recommend the early use of oral steroids in the treatment algorithm of BP [2]. Several reports have showed greater recovery in nerve function with high dose oral steroids with or without anti-viral medication compared to placebo. In a recent double-blind randomized controlled study, Sullivan et al. reported that early treatment with prednisolone significantly improved the potential of complete recovery, up to 94.4% within 9 months compared to 81.6% for patients not taking steroids [6]. These findings have been further supported by a recent Cochrane review [20]. Our study did not include a control arm of subjects treated conservatively with medical therapy alone. Nonetheless, part of our data can be compared to outcomes of conservative management available in the literature. For this comparison, it would be most appropriate to include the subset of our patients who had both oral steroids pre-operatively and a follow-up of at least 6 months. Eleven of our 36 subjects fill these criteria. Of these, 45.5% (5/11) achieved HB grade 2 or better and 100% HB 3 or better. Control groups of previous studies have had recovery to HB 2 or better between 41.7 and 65% and improvement to HB 3 or better between 81.1 and 94.4% [9, 14, 19]. This comparison may suggest that delayed decompression helps avoid severe persistent facial paralysis of HB grade 4 or worse. In turn, it would help reduce the most undesired sequelae of BP, namely incomplete eye closure and the resulting ocular complications.

The pathophysiologic mechanism of BP and findings at surgery by some authors may help explain the role of delayed facial nerve decompression in refractory BP. Although a clear causative mechanism remains to be determined, viral pathogens, notably herpes simplex virus (HSV) have been identified as potential triggers causing nerve inflammation and conduction dysfunction [21]. The inflammatory response occurring along the facial nerve appears to induce an intrinsic compression at its narrowest anatomical passage in the labyrinthine segment. Supporting this theory, facial nerve inflammation and edema has been reported as common intraoperative findings in BP patients [9, 15]. Interestingly, Bodenez et al. consistently noted inflammatory changes of the facial nerve in all of their 13 patients who underwent delayed surgical decompression, even in those operated 4 months following onset of facial paralysis [15]. Yanagihara et al. also noted edematous swelling of the facial nerve in most of their patients up to 3 months after onset [9]. Based on these findings and the results of our study, we could hypothesize that the inflammatory process may persist up to or even beyond 90 days following BP. Alternatively, our results may also be suggestive of a fibrotic remodeling process following inflammation that can maintain intrinsic compression on the nerve, which would then be relieved following surgical decompression [22].

A mild correlation was found between length of follow-up and improvement in facial nerve function. This finding corroborates the clinical observation that facial nerve function can still improve up to 6 months after an episode of Bell’s palsy [5].

The optimal surgical approach to decompress the facial nerve is still debated. According to Cannon et al., facial nerve decompression should be achieved within 2 weeks of onset of BP using the middle fossa approach. Their study revealed improvement rates of 71% with low morbidity [10]. Other studies have reported good results and improved nerve recovery with the transmastoid approach [9, 13, 23–25]. In our study, all patients underwent transmastoid surgical decompression surgery as it was the preferred approach of the senior neurotologist (JJD).

Our study shows several limitations including its retrospective nature, a low proportion of patients having had pre-operative ENoG and EMG evaluations, and the absence of a control group treated with medical management alone. Electrophysiological assessment of facial nerve function was not available for all patients due to limited resources in primary and secondary centers in addition to delayed referral to our tertiary care center. We were not able to confirm that all our patients had >90% denervation prior to surgery. There is therefore a risk of overestimating the impact of delayed decompression. Moreover, although considered the most commonly used tool for the clinical assessment of facial nerve function, the HB grading scale has inherent limitations regarding its subjectivity, reliability, and longitudinal applicability [26]. These limitations of the HB scale were partially offset by having all the patients assessed by the same surgeon pre and post-operatively. Finally, the risk of hearing loss associated with facial nerve decompression is an important consideration to discuss with patients prior to surgical intervention. Significant traumatic sensorineural hearing loss is estimated between 2 and 5% [19]. In the present study, hearing assessment data was not consistently available, and no conclusions could be drawn regarding the rate of postoperative hearing loss.

## Conclusion

The present study offers additional evidence that may support the role of delayed decompression surgery for BP. We recommend facial nerve decompression for BP within the first 2 weeks when indicated. Nonetheless, our results suggest that patients with severe BP may benefit from decompression surgery up to 90 days after the onset of symptoms in the absence of an opportunity to proceed earlier to surgery. Further investigation is still required to confirm our findings and to better define the role of delayed surgical intervention in the treatment algorithm of BP, especially for patients with persistent paralysis beyond 3 months.

## Abbreviations

BP: Bell’s palsy
EMG: Electromyography
ENoG: Electroneuronography
HB: House-Brackmann
